# Transcriptome data analysis provides insights into the conservation of *Michelia lacei*, a plant species with extremely small populations distributed in Yunnan province, China

**DOI:** 10.1186/s12870-024-04892-1

**Published:** 2024-03-19

**Authors:** Yang Liu, Lei Cai, Weibang Sun

**Affiliations:** 1 Yunnan Key Laboratory for Integrative Conservation of Plant Species With Extremely Small Populations/ Key Laboratory for Plant Diversity and Biogeography of East Asia, Kunming Institute of Botany, Chinese Academy of Sciences, Kunming, Yunnan 650201 China; 2 University of Chinese Academy of Sciences, Beijing, 100049 China

**Keywords:** *Michelia lacei*, Genetic diversity, Population dynamics, Environmental adaptability, Conservation genomics

## Abstract

**Background:**

*Michelia lacei* W.W.Smith (Magnoliaceae), was classified as a Plant Species with Extremely Small Populations (PSESP) by the Yunnan Provincial Government in both action plans of 2012 and 2021. This evergreen tree is known for its high ornamental and scientific value, but it faces significant threats due to its extremely small population size and narrow geographical distribution. The study aims to understand the genetic structure, diversity, and demographic history of this species to inform its conservation strategies.

**Results:**

The analysis of transcriptome data from 64 individuals across seven populations of *M. lacei* identified three distinct genetic clusters and generated 104,616 single-nucleotide polymorphisms (SNPs). The KM *ex-situ* population, originating from Longling County, exhibited unique genetic features, suggesting limited gene flow. The genetic diversity was substantial, with significant differences between populations, particularly between the KM lineage and the OTHER lineage. Demographic history inferred from the data indicated population experienced three significant population declines during glaciations, followed by periods of recovery. We estimated the effective population size (Ne) of the KM and OTHER lineages 1,000 years ago were 85,851 and 416,622, respectively. Gene flow analysis suggested past gene flow between populations, but the KM *ex-situ* population showed no recent gene flow. A total of 805 outlier SNPs, associated with four environmental factors, suggest potential local adaptation and showcase the species' adaptive potential. Particularly, the BZ displayed 515 adaptive loci, highlighting its strong potential for adaptation within this group.

**Conclusions:**

The comprehensive genomic analysis of *M. lacei* provides valuable insights into its genetic background and highlights the urgent need for conservation efforts. The study underscores the importance of *ex-situ* conservation methods, such as seed collection and vegetative propagation, to safeguard genetic diversity and promote population restoration. The preservation of populations like MC and BZ is crucial for maintaining the species' genetic diversity. *In-situ* conservation measures, including the establishment of *in-situ* conservation sites and community engagement, are essential to enhance protection awareness and ensure the long-term survival of this threatened plant species.

**Supplementary Information:**

The online version contains supplementary material available at 10.1186/s12870-024-04892-1.

## Background

Over thousands of years, species have acquired their ecological niches through adaptations to local phenological conditions and competition with other species [1–3]. With the advent of the anthropocene, the balance of competition among species has been disrupted. Unrestricted anthropogenic resource acquisition and environmental degradation have led to the extinction of thousands of species [4–6]. Furthermore, even where species have not gone extinct, populations have often experienced significant declines. Currently, more than 1 million of the world's species are at high risk of extinction [7]. For threatened species, long-term anthropogenic disturbances result in habitat fragmentation and reduced pollinator populations, leading to decreased inter-population communication and hindering the preservation of genetic diversity within species [8]. Moreover, prolonged intra-population gene flow due to human interference can lead to the accumulation of detrimental alleles [9, 10]. Under rapid climate change scenarios, isolated populations will be further disadvantaged in adapting to future environmental conditions [4]. Moreover, habitat fragmentation diminishes the population sizes of threatened species, resulting in heightened genetic drift, diminished heterozygosity among individuals, and reduced adaptability [11]. In order to effectively prevent the extinction of species due to genetic factors, it is therefore necessary to provide recommendations on the conservation of species at the molecular level.

The development of conservation genomics has greatly aided our understanding of the genetic background of threatened species, providing valuable insights for their conservation at the molecular level [12–14]. As high-throughput sequencing technology advances, the molecular markers utilized for analysis have become increasingly precise and specific. This enables us to acquire comprehensive data on gene loci for studies on gene function, population structure, genetic diversity, population history, and genetic vulnerability [15–18]. The use of the sequenced transcriptome as a reference genome allows the identification of accurate and comprehensive transcript sequences and expression information, making it a commonly used tool in conservation genetics analysis [19]. By analyzing high-quality transcriptome data, we can study the adaptive evolution of species, compare sequence functional information with the known genomes of other species, and compare gene expression differences within the same species [20, 21].

*Michelia lacei* W.W.Smith is an evergreen tree belonging to the family Magnoliaceae. There are more than 120 species of Magnoliaceae in China, mainly distributed in the temperate, subtropical, and tropical regions, particularly in the southwest and south of the country [22, 23]. The populations of *M. lacei* in China are mainly found in southeastern and western Yunnan province, in lower montane evergreen bread-leaved forest at elevations of 1,000 to 1,800 m [24]. However, no population have been found in the area between western and southeastern Yunnan. The species is also reported in Vietnam and Myanmar [24, 25]. Of the approximately 40 species in the genus of *Michelia*, fifteen (including *M. lacei*) are listed as Endangered (EN) in the China Biodiversity Red List-Higher Plants Volume (2020). Additionally, *M. lacei* was listed as one of the 101 plant species in urgent need of rescue and protection in the "List of Yunnan Protected Plant Species with Extremely Small Populations (2021)" [26]. Several researchers have conducted conservation biology studies on threatened species in the Magnoliaceae using datasets of SNPs (e.g. [18, 27–29]), but researches on *M. lacei* have been limited to population surveys and habitat assessment [24]. Therefore, this study aims to explore the conservation genetics of *M. lacei* by analyzing transcriptome data.

The objectives of this study are to (a) understand the current genetic structure and genetic diversity among different populations of *M. lacei* in China by using molecular markers; (b) investigate the gene flow that occurred during the formation of the current population structure of *M. lacei*, describe the demographic history of the species and suggest how it was affected by climate change; (c) explore the response of *M. lacei* to the current environment by screening for outlier SNPs; and (d) provide more targeted conservation recommendations for *M. lacei* based on the results obtained from the SNP data.

## Results

### Sequencing results and SNP calling

Transcriptome sequencing was performed on 64 leaf samples, generating approximately 6G data per sample, and resulting in a total of approximately 3.594 billion reads. Each sample was aligned to the whole genome of *Magnolia sinica* (also commonly use the name of *Manglietiastrum sinicum* in China), with alignment rates ranging from 97 to 99% (Table S1), indicating high alignment rates. After filtering, a total of 104,616 SNPs were obtained from the VCF, forming Dataset 1. Subsequently, 15,360 SNPs were obtained after Dataset 1 was subjected to 4DTv screening, forming Dataset 2 (putatively neutral dataset). After LD filtering was performed on Dataset 1, a total of 42,481 SNPs remained, forming Dataset 3.

### Population structure and genetic diversity

**Fig. 1:**
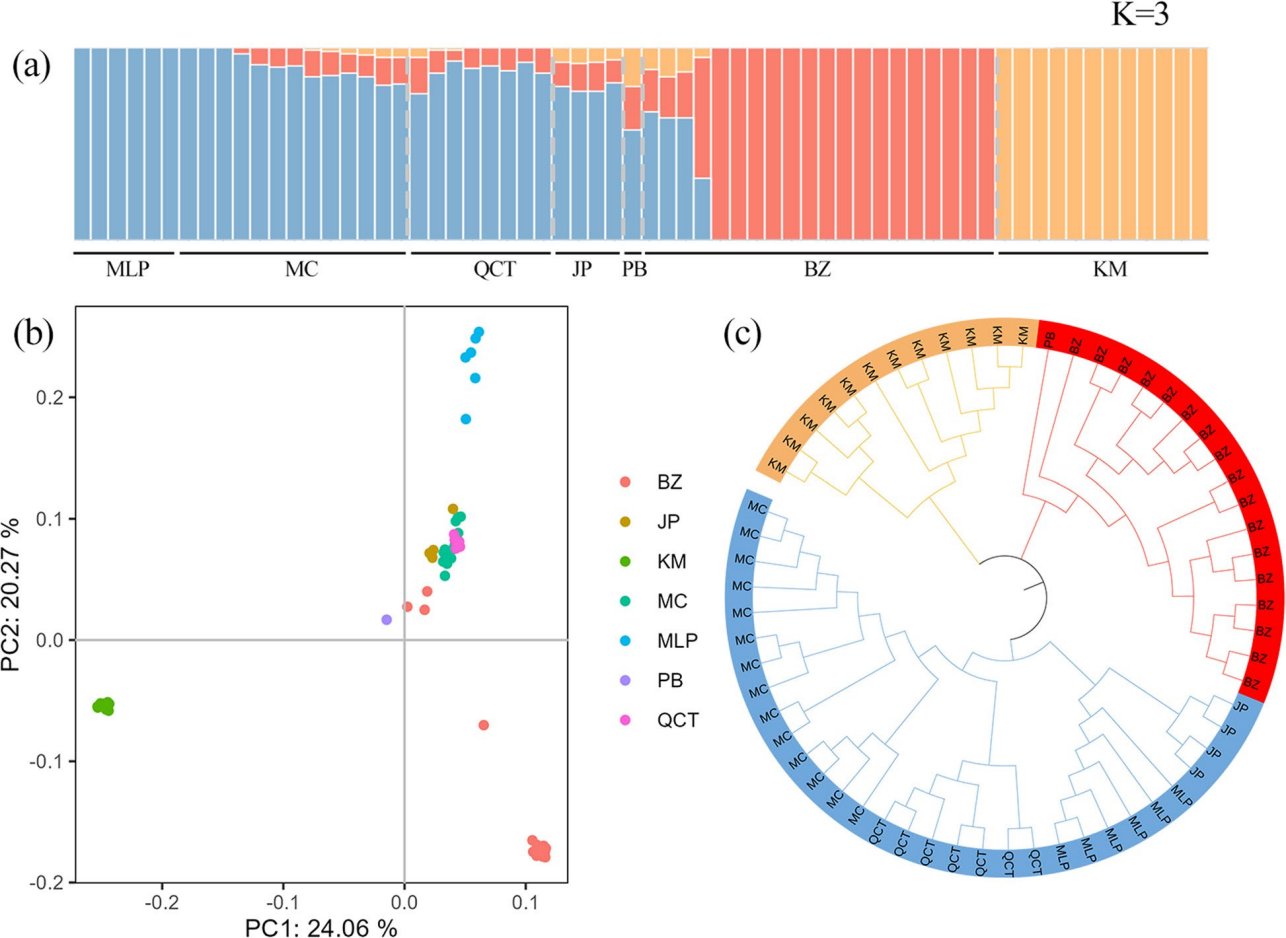
Population structure of *Michelia lacei*. **a** Genetic structure obtained in Dataset 3 when K = 3. Different colors represent different genetic backgrounds. **b** Principal components analysis scatterplots based on the PCA results obtained from Datasets 3, where each point represents one plant. **c** Maximum-likelihood phylogenetic tree constructed based on Dataset 2

To analyze the genetic structure of *M. lacei*, ADMIXTURE was used to analyze Datasets 2 (Fig. S1) and 3 (Fig. 1a). The best results were obtained when K = 3, with CV values of 0.60492 and 0.49150 for Datasets 2 and 3, respectively (Table S2). It was clear that the KM *ex-situ* population was distinct from the others. A clear geographical division exists, as the trees of KM *ex-situ* population originated in Longling County, Baoshan in western Yunnan, while the other populations were located in southeastern Yunnan. To further investigate the genetic structure, PCA analysis was also performed on Datasets 2 (Fig. S2) and 3 (Fig. 1b) using EIGENSOFT. The first and second principal components (PC1 and PC2) explained more than 20% of the variance in both datasets, indicating a good explanation of the data variability. In the PCA plots, it was also evident that KM *ex-situ* population was distinct from other populations, while populations of MC, QCT, JP, PB, and BZ clustered together, suggesting a correlation or shared characteristics among these populations and agreeing with the ADMIXTURE results. The reconstructed phylogenetic neighbor-joining tree suggested that the samples could be grouped into three genetic units (Fig. 1c). The six populations in southeastern Yunnan mainly consist of two genetic backgrounds, with genetic mixing between them. The KM *ex-situ* population is relatively pure.

**Fig. 2:**
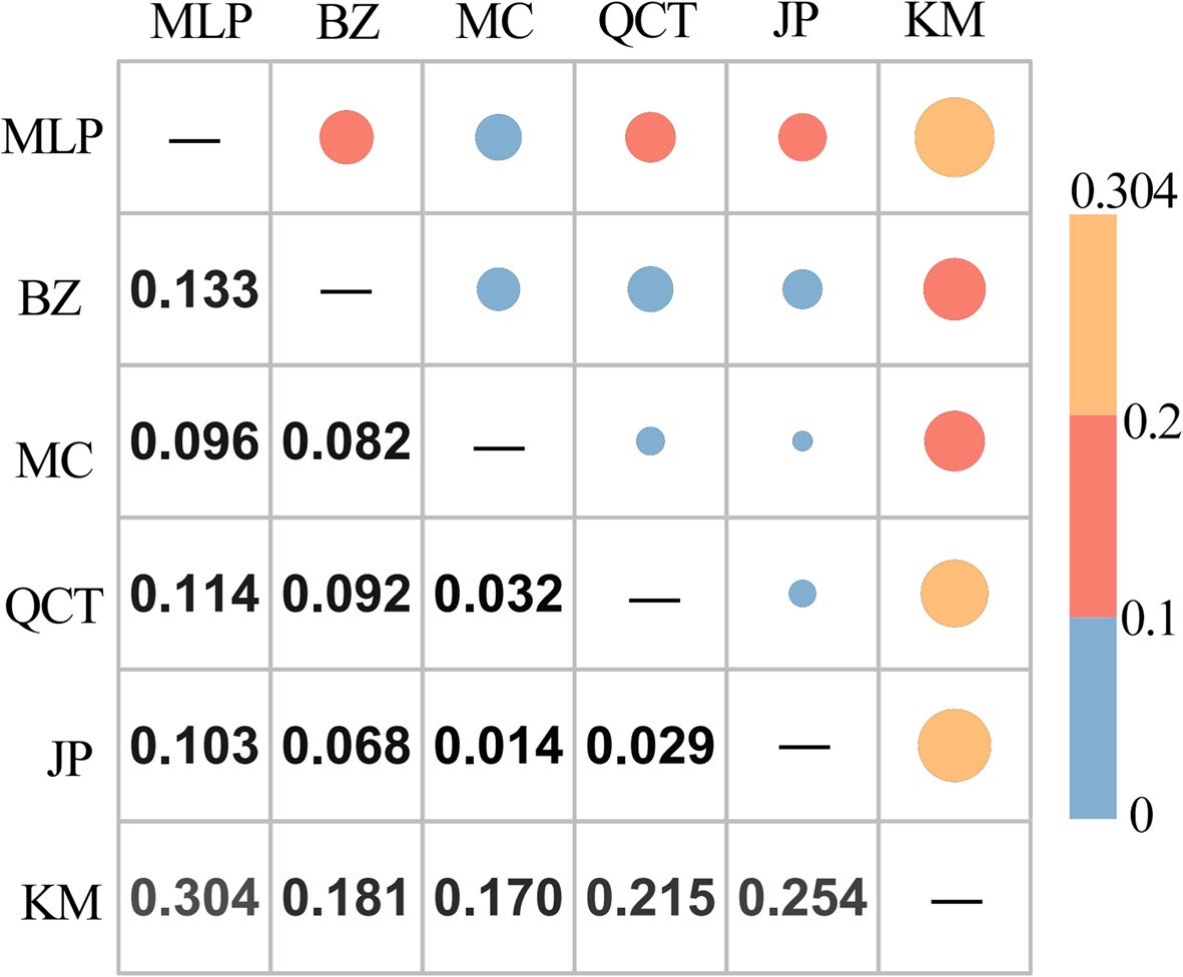
Genetic distances (*F*_ST_ values) between *Michelia lacei* populations. The larger the circle, the larger the value, which ranges from 0–0.304

All populations except PB were included in the calculation to assess genetic diversity. Computing values of genetic diversity based on Datasets 1 (Table 1) and 2 (Table S3) maintain essentially similar trends. For Dataset 1, the overall π ranged from 0.157 (KM) to 0.286 (MC). The *H*_O_ ranged from 0.248 (MC) to 0.360 (KM), while *H*_E_ within populations ranged from 0.556 (JP) to 0.676 (BZ). The values of *F*_IS_ ranged from 0.150 (KM) to 0.298 (MLP), and Tajima's *D* values ranged from 0.536 (JP) to 1.246 (KM). The average π, *H*o, *H*_E_, *F*_IS_, and Tajima's *D* calculated for all populations were 0.298, 0.190, 0.702, 0.358, and 1.090, respectively (Table 1). Population differentiation estimates (*F*_ST_) were also derived from Datasets 1 (Fig. 2) and 2 (Table S4). In Dataset 1, the smallest *F*_ST_ value was 0.014, observed between JP and MC, while the largest *F*_ST_ value was 0.304, observed between MLP and KM. The Mantel test results indicate a strong correlation between geographical distance and genetic distance (IBD, *R*^2^ = 0.501, *p* = 0.001), as well as environmental distance and genetic distance (IBE, *R*^2^ = 0.519, *p* = 0.001). These findings suggest that differences in geographical and environmental factors can have a significant impact on the genetic differentiation between individuals (Fig. S3).

**Table 1:** Measures of genetic diversity for 63 *Michelia lacei* individuals from Dataset 1. N, number of individuals in the population; π, nucleotide diversity; *H*_O_, observed heterozygosity; *H*_E_, heterozygosity within populations; *F*_IS_, inbreeding coefficient; Tajima's *D*, neutrality test statistics

Population	N	π	*H*_O_	H_E_	F_IS_	Tajima's *D*
MLP	6	0.189	0.264	0.595	0.298	0.624
BZ	20	0.267	0.269	0.676	0.161	0.810
MC	14	0.286	0.248	0.664	0.244	0.731
QCT	7	0.276	0.286	0.624	0.208	0.576
JP	4	0.247	0.322	0.556	0.221	0.536
KM	12	0.157	0.360	0.570	0.150	1.246
Overall	63	0.298	0.190	0.702	0.358	1.090

### Population history inference and gene flow

**Fig. 3:**
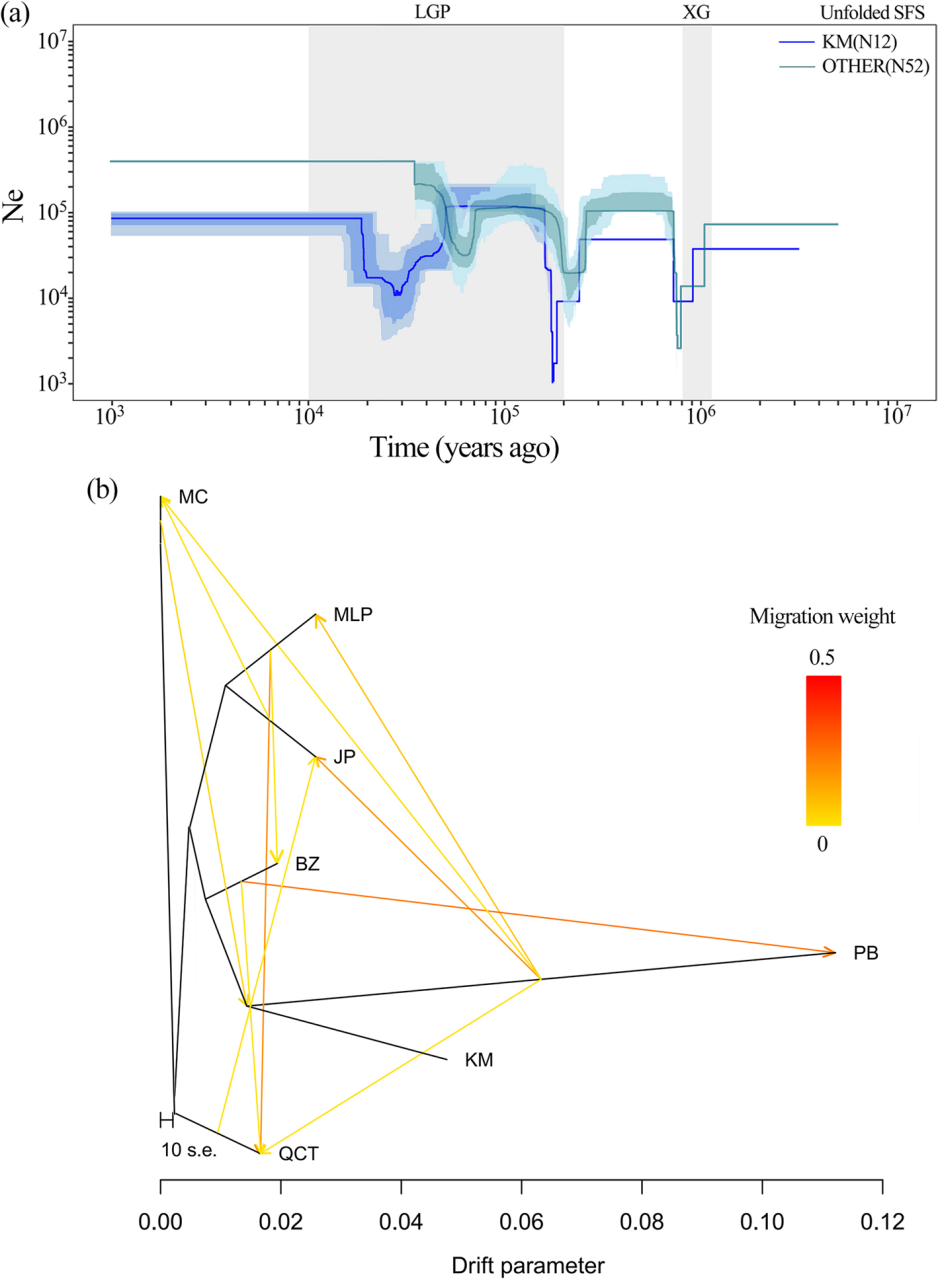
**a** Population history was inferred for two *Michelia lacei* lineages (KM, OTHER) based on Unfolded SFS. The 95% confidence interval for the estimated effective population size is shown in a light color, and the thick lines represent the median values. The light grey areas represent different glaciation events during the Pleistocene (XG, Xixiabangma Glaciation; LGP, Last Glaciation Period). **b** Relationships and gene flow between *M. lacei* populations, depicted as the maximum-likelihood tree produced in Treemix. The direction of gene flow is indicated by an arrow

The population history of *M. lacei* was inferred using Stairway Plot 2. The demographic histories were calculated for both Folded and Unfolded SFS. In the analysis using Folded SFS (Fig. S4), the KM lineage experienced two population declines, one occurring around 0.4–0.2 million years ago (Mya) and another during the Last Glacial Maximum (LGM, 26–19 Kya) [30]. The OTHER lineage experienced a population decline during the Xixiabangma Glaciation (XG, 1.2–0.8 Mya) [31]. In the analysis using Unfolded SFS (Fig. 3a), both lineages experienced three population declines. In addition to the declines observed in the Folded SFS analysis, the KM lineage also experienced a decline around 0.8–1 Mya. Apart from this, the population histories were similar to those in the Folded SFS analysis. In both scenarios, after the Last Glacial Period (LGP, 110–10 Kya) [32], the population sizes remained relatively stable. From Stairway Plot 2, we predicted the effective population size (Ne) 1,000 years ago for the KM and OTHER lineages to be 85,851 and 416,622, respectively. The number of extant individuals today is far less than was predicted from this analysis, indicating that *M. lacei* numbers have been in constant decline since 1000 years ago.

When calculating migration events in gene flow, the best results were obtained when the migration event value was set to 12 (Fig. 3b). The output from TreeMix analysis showed arrows representing the direction of gene flow, with darker colors indicating stronger levels of gene flow. The strongest gene flow was predicted to have been mainly from BZ to PB, from MLP to QCT, and from PB to JP. Other gene flow between populations was also primarily observed within the six populations in southeastern Yunnan. No recent gene flow between KM (originating from Longling in west Yunnan Province, China) and other populations was observed in the TreeMix result.

### Detection of outlier SNPs and GO annotations

**Fig. 4:**
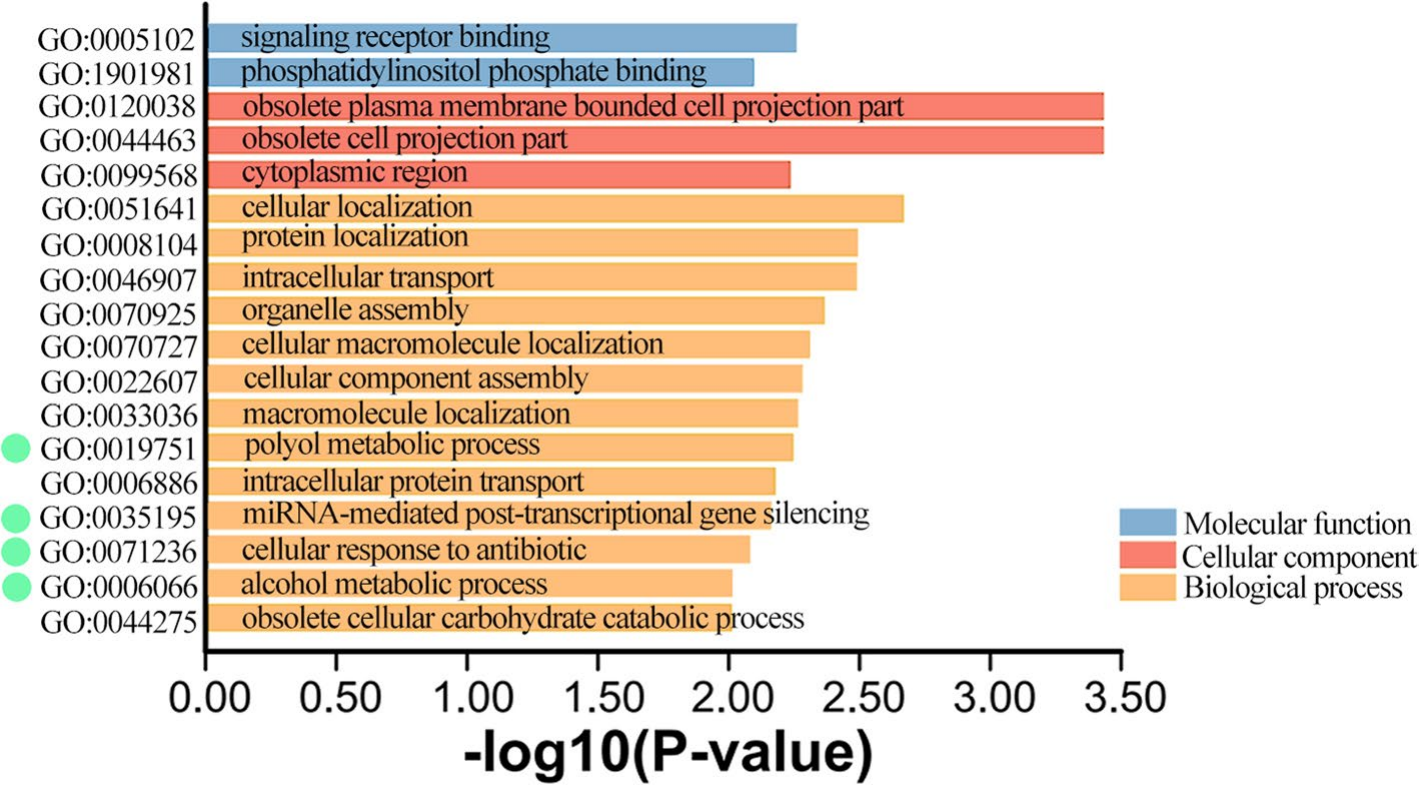
GO enrichment analysis of environment-associated genes (the combined data from BAYESCENV and RDA) in *Michelia lacei* (*P* < 0.01). Light blue, light red, and light orange represent “molecular function”, “cellular composition”, and “biological processes”, respectively. Green circles represent gene expression associated with environmental stress

Two methods, BayeScEnv and RDA, were used to filter SNPs related to the selected environmental factors (BIO3, BIO7, BIO13, BIO14). Following BayeScEnv filtering, a total of 173 SNPs associated with the above four environmental factors were retained, with 51 SNPs related to BIO3, 46 SNPs related to BIO7, 15 SNPs related to BIO13, and 129 SNPs related to BIO14. RDA detected a total of 636 SNPs related to the four environmental factors, namely 168 (BIO3), 248 (BIO7), 112 (BIO13), and 108 (BIO14) (Table S5). After merging and removing duplicates, a total of 805 SNPs related to climate adaptation were identified from both methods. These five populations exhibit variability in climate adaptation loci, specifically BZ (515), QCT (393), JP (448), MC (494), and MLP (438). To identify the genes with which these outlier loci were associated, the SNPs were matched to the annotation file from the *Magnolia sinica* genome, resulting in the localization of 666 genes. Gene Ontology (GO) enrichment analysis was conducted to identify gene functions, and a total of 79 genes showed significance (*P* < 0.05), including 18 genes with *P* < 0.01 (Fig. 4, Table S6). Two genes were associated with “molecular function” (MF), three genes were associated with “cellular component” (CC), and the remaining 13 genes were associated with “biological process” (BP).

## Discussion

### Genetic structures and genetic diversity of *M. lacei*

Geographic distance is considered to be an important factor in genetic differentiation between populations. Due to geographic isolation, gene flow between populations in different regions may be limited or absent, resulting in reduced genetic exchange. This can lead to the accumulation of genetic frequency differences, and thus genetic differentiation [33–35]. Furthermore, a significant biogeographical boundary called the "Tanaka-Kaiyong Line" (TKL) separates the populations in western Yunnan from those in southeastern Yunnan [36–38], and previous studies have found significant phylogeographic differentiation between *Sophora davidii* populations on opposing sides of the TKL [32].

In this study, three methods were used to analyze the genetic structure of *M. lacei*. All of these methods suggested three genetic units existed within the study individuals. Populations in the southeastern Yunnan province, with the exception of MLP population, exhibited a genetic structure that included multiple genetic backgrounds. The PB, BZ, MC, and QCT populations were genetically close, and both the structure and PCA analyses showed similar genetic compositions and evidence of gene flow between these populations. The low *π* value (0.157) in the KM *ex-situ* population suggests the potential for close kinship between individuals, possibly due to the same maternal parent or adjacent plant, resulting in low levels of nucleotide diversity. The values of Tajima's *D* for each population were more than 0, which indicates the presence of an excess of intermediate frequency alleles in the population, which may result from either natural selection or genetic drift. This suggests that certain genes provide an advantage for environmental adaptation, leading to an increased frequency of associated gene expression in *M. lacei.* [39–41]. *F*_ST_ values exceeding 0.25 between different populations within the same species indicate significant genetic differentiation [42]. In this study, the degree of the *F*_ST_ (0.170–0.304) between KM and other populations was relatively higher. Because KM *ex-situ* population originated from Longling in west Yunnan province, we speculate that long distances lead to increased genetic differentiation between populations over time.

The genetic diversity of *M. lacei* is high relative to several other threatened species, indicating that the endangered status of this species is not caused by inbreeding depression [17, 18]. Combining these data with the findings from our field work and observations, we speculate that the population size decline of *M. lacei* in recent generations is due to human activities.

### Demographic history and gene flow reflect the formation of genetic patterns in *M. *lacei

Analysis of gene flow can determine the extent of genetic exchange between populations and allow prediction of the genetic connectivity between different populations [43, 44]. This study has shown that the KM *ex-situ* populations of *M. lacei* has not received any recent genetic flow from other populations. The lack of gene flow from other populations accelerates the rate of genetic loss, reduces population adaptability and survival capacity, and increases the risk of population extinction in the long term [45–48]. In southeastern Yunnan, genetic flow analysis in *M. lacei* populations indicates that past gene flow has occurred. However, due to human activities and habitat degradation, populations have become more isolated. Consequently, the decrease in pollinators has led to reduced gene flow between different populations [17, 18].

Studying the population history dynamics of a species can enhance our understanding of its origin, population expansions, and the differentiation processes between populations [49, 50]. Based on geographical differences between populations, two lineages (KM and OTHER) were identified. The results of our population history inference suggested that both lineages have experienced three bottleneck events. These bottleneck events occurred around XG, LGP, and LGM, respectively. Both the KM and OTHER lineages had a significant population size decline during XG (1.2–0.8 Mya). After the glaciation, the Ne recovered and the species entered a relatively stable period. However, just before entering the LGP, a second, more severe bottleneck event occurred, with the Ne of the KM *ex-situ* population being almost reduced to zero on the arrival of the glaciation. During the long glaciation period, there were several interglacial periods (warm periods between glaciations), which allowed a temporary recovery of Ne in different populations [32]. During the LGM, the Qinghai-Tibet Plateau was significantly affected by the glaciation, covering Tibet, Qinghai, and western Sichuan [30]. The KM *ex-situ* population was therefore more significantly affected by the LGM than the OTHER lineage, and experienced a further bottleneck effect during this period. After that, the Ne of all populations remained relatively stable for approximately ten thousand years. Other studies have also shown that the last glaciation has influenced the formation of different regional lineages in the eastern TKL, and *Sophora davidii* shows a similar pattern of bottleneck events to *M. lacei* [51]. Upon investigation, it was discovered that the actual number of *M. lacei* individuals is significantly lower than the calculated result. Our field investigation suggests that this discrepancy is primarily attributed to the following factors: (1) Construction of roads and buildings; (2) Cultivation of cash crops under the forest, impeding the natural regeneration of *M. lacei*; (3) Utilization of wood by local communities. These various factors are likely to have played a part in the significant decrease in the population of *M. lacei* individuals over the past millennium.

### Local adaptation of *M. lacei* populations

In addition to geographic isolation, different environmental factors also play an important role in shaping the genetic background of different populations [20, 21, 52]. BIO7 has demonstrated the most significant impact on the survival of *M. lacei* among the four environmental factors. In all field populations, BZ has the highest number of adaptive loci, totaling 515, while QCT was the least frequent, with only 393. GO enrichment analysis revealed that several genes with a significance level of *P* < 0.01 were associated with biological processes (BP).

Furthermore, analysis of gene functions revealed that the significant expression of genes related to “polyol metabolic process” (GO:0019751), “miRNA-mediated post-transcriptional gene silencing” (GO:0035195), “cellular response to antibiotic” (GO:0071236), and “alcohol metabolic process” (GO:0006066) indicated that the plants were undergoing specific physiological processes, responding to stress, or engaging in specific metabolic regulation. Their expression may be a response of the plants to adapt to environmental changes or cope with biotic stresses.

In addition, four individuals from Longling, Baoshan, cultivated in the Kunming Botanical Garden (KBG) have been able to flower and bear fruits normally, and the other eight plants in KM *ex-situ* population are growing also in very good condition. This suggests that the species is able to thrive in the climatic conditions of Kunming, which is located much further north from Longling in West Yunnan. It also demonstrates its strong adaptability.

### Guidelines for the conservation of *M. lacei*

The Magnoliaceae is one of the most important groups of plant species in China's subtropical evergreen broad-leaved forests [53]. However, more than 76 species in the family, are currently assessed as threatened in China, accounting for more than 50% of the total species in Magnoliaceae in the country [54]. Of these, *M. lacei,* which has been prioritized as a PSESP in China, is in urgent needs of rescue protection. Therefore, based on the results shown in this study, we put forward the following suggestions for the conservation of *M. lacei*: (1) Seeds from individuals with varying genetic backgrounds should be gathered from wild populations to facilitate the reproduction of seedlings. This will enable the *ex-situ* conservation of seedlings from different genetic backgrounds in the KBG, thereby preserving the genetic diversity of the species. Specifically, populations such as MC and BZ, which exhibit distinct genetic backgrounds, should receive particular attention. (2) By collecting vegetative materials of individuals in KM *ex-situ* population to propagate young plants by cuttings or by tissue culture, and the rooted cuttings or young plants can be used for the population reinforcement and population restoration in its originated habitats in Longling of west Yunnan province. The *F*_IS_ of the KM *ex-situ* population was the lowest of all the populations. However, to prevent the accumulation of harmful genes resulting from selfing and inbreeding in the *ex-situ* individuals' seeds, it is advisable to avoid using seedlings propagated from seeds of KM *ex-situ* individuals for natural population restoration. (3) All the original habitats of *M. lacei* outside the nature reserves should be well protected by establishing the *in-situ* conservation sites. For scattered individuals along the roadsides and by the villages, information dissemination projects should be conducted together with local communities for increasing the protection awareness of general publics.

## Conclusions

The study provides a comprehensive genomic analysis of *M. lacei*, a plant species with extremely small populations in Yunnan Province, China. Through the use of transcriptome data, the research has elucidated the genetic structure and diversity within and among populations, revealing three distinct genetic clusters. The findings suggest that historical gene flow has shaped the current genetic landscape, with the KM *ex-situ* population showing a particularly unique genetic signature. The study also highlights the adaptive potential of *M. lacei* populations, as evidenced by the identification of outlier SNPs associated with environmental factors, which could be crucial for the species' resilience in the face of environmental challenges.

The demographic history inferred from the analysis indicates that *M. lacei* has experienced significant population declines during past glaciations, with a stabilization of population size around 10,000 years ago. However, the current population size is alarmingly low, with only 52 individuals known in the wild in China. This decline is likely due to human activities, including habitat fragmentation and degradation.

This study highlights the significance of conservation initiatives for *M. lacei*, stressing the necessity of *ex-situ* conservation methods, such as seed gathering and vegetative propagation, to safeguard genetic diversity and promote population restoration. The preservation of populations such as MC and BZ would guarantee the highest possible conservation of genetic diversity in *M. lacei*. Additionally, we calls for *in-situ* conservation measures, including the establishment of mini-reserves and community engagement to enhance protection awareness. The insights gained from this study not only contribute to the conservation of *M. lacei* but also serve as a valuable reference for the conservation of other threatened species within the Magnoliaceae.

## Materials and methods

### Sample collection and RNA extraction

**Fig. 5:**
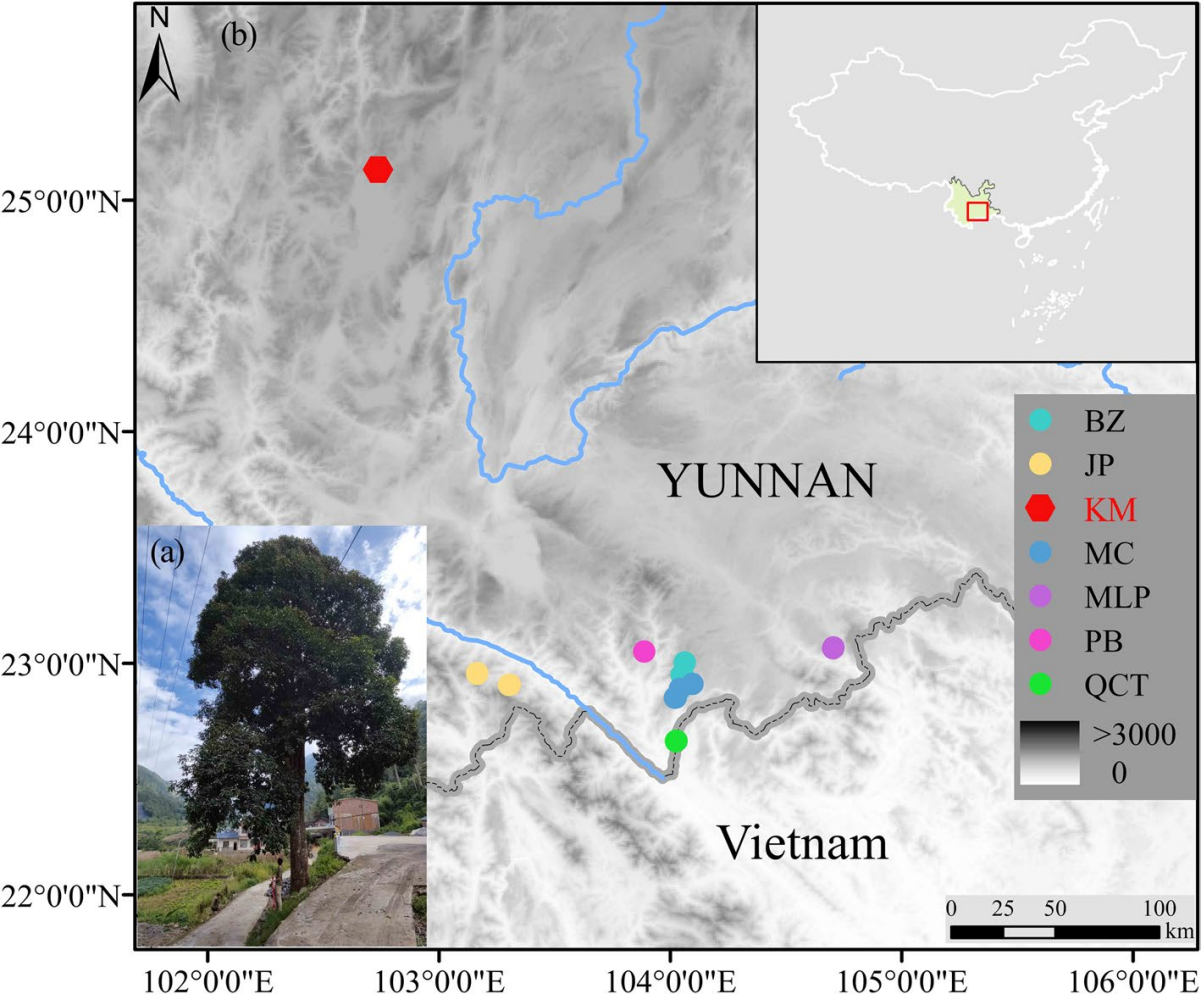
The distribution of *Michelia lacei* populations collected in this study (**a**) A *M. lacei* tree located in Bazhai Town, Maguan County with a brand of ancient and rare tree in Yunnan Province. **b** Geographic distribution of six (BZ, JP, MC, MLP, PB, QCT) wild populations and one (KM) *ex-situ* population. Note: Different colored circles represent different populations (Table S7), black dotted lines indicate national borders, and blue lines represent rivers (China standard map GS (2019) 1822, border without modification), Elevation (www.worldclim.org)

Based on botanical field surveys of *M. lacei* in China, only six populations of PB, MLP, BZ, MC, QCT and JP were found, comprising a total of 52 individuals in the wild. Additionally, there are 12 *ex-situ* cultivated individuals from Kunming Botanical Garden (KM), which were propagated from seeds collected from the individuals in Longling County of west Yunnan in 1987. Unfortunately, we did not find wild individuals near Longling despite several recent field investigations. A total of 64 individuals of all available populations were collected for this study (Fig. 5, Table S6). We collected voucher specimens and recorded information about the flowers, fruits, leaves, and other characteristics of the plants in the field. Our samples of *M. lacei* were verified by Lei Cai as matching the descriptions in the *Flora of China*. The voucher specimens were deposited at the Herbarium, Kunming Institute of Botany, Chinese Academy of Sciences (code YL2022001–YL2022007, Table S6).

Fresh and insect-free leaves were collected from plants and were placed directly into liquid nitrogen tanks for preservation in the field. Upon returning to the laboratory, the samples were stored at -80 °C. To extract RNA from the leaves, leaves were ground in liquid nitrogen, and ≤ 100 mg of ground sample was added to centrifuge tubes. The modified CTAB method was used to extract RNA from each collected sample [55]. Agarose gel electrophoresis was performed to check for sample degradation, and the purity of the obtained RNA was assessed using a Nanodrop. RNA was quantified accurately using Qubit. After RNA samples had passed the quality control, random fragmentation was performed using a Covaris ultrasonic disruptor at BenaGen (Wuhan, China). RNA enrichment was then conducted, and the first strand of cDNA was synthesized using the extracted RNA as a template. Subsequently, the RNA was degraded, and the second strand of cDNA was synthesized using dNTPs. PCR amplification was performed, and after library construction, the samples were subjected to paired-end sequencing on the Illumina Novaseq 6000 platform, which provides higher sequencing data quality compared to single-end sequencing.

The raw data obtained were filtered using fastp v.0.21.0 [56] to remove reads with an N base content exceeding 5%, those with a low-quality base count reaching 50%, those contaminated with adapters, and duplicate sequences caused by PCR amplification. Subsequently, the filtered data were subjected to quality control using FastQC v.0.11.9 [57]. The results indicated good data quality, and the obtained clean reads were then used for downstream analysis.

### Sequence alignment and screening of SNPs

To accurately identify SNPs in the transcriptome data, it is necessary to have a reference genome for sequence alignment. In this study, the chromosome-level genome of *Magnolia sinica* (BioProject ID PRJNA774088), was used as the reference genome. SAMtools v.1.9 [58] and BWA-MEM v.0.7.17 [59] were used to index the whole genome for subsequent alignment. The filtered clean reads were aligned to the reference genome of *M. sinica* using BWA-MEM v.0.7.17, and the alignment results were stored in binary format (bam) files. To ensure the accuracy and reliability of the data, duplicate reads were removed using the MarkDuplicates tool in Picard v.2.20.3 (http://broadinstitute.github.io/picard/). The alignment rate of reads was calculated using SAMtools' flagstat, where a higher alignment rate indicated higher alignment efficiency and sequencing data quality. The bam files were converted into variant call format (vcf) files containing genomic variation information, using HaplotypeCaller in GATK v.4.2.0 [60]. Then, the vcf files of all individuals were combined into one vcf file using CombineGVCFs in GATK v.4.2.0. Population-level variant detection was performed using GenotypeGVCFs, and the detected variants were filtered using VariantFiltration with the following parameters "window 35, cluster 3, FS > 30.0, QD < 2.0". Filtered SNPs were extracted using SelectVariants. To obtain high-quality SNPs, the dataset was then further filtered using VCFtools v.0.1.13 [61] with max missing < 0.5 and minor allele frequency (MAF) < 0.05. This resulted in a comprehensive dataset of SNPs (Dataset 1) suitable for subsequent analysis. Dataset 1 was subjected to fourfold degenerate sites (4DTv) filtering using SnpEff v.4.0 [62] to generate a collection of putatively neutral loci (Dataset 2).

### Genetic structure and genetic diversity

To reduce redundant information introduced by highly correlated SNPs, Dataset 1 was subjected to Linkage Disequilibrium (LD) filtering using PLINK v.1.90 [63] before analysis. The parameters were set as "-indep-pairwise 50 5 0.4", resulting in a subset of SNPs constituting Dataset 3.

ADMIXTURE v.1.3.0 [64] was used to infer the population structure of *M. lacei* based on Datasets 2 and 3. The value of K was set to range from 2 to 5. The optimal K value for population structure partitioning was determined by selecting the value associated with the lowest cross-validation error. The results were visualized using Pophelper (http://pophelper.com/). To validate the population structure results, two additional methods were employed. First, principal component analysis (PCA) was performed on Datasets 2 and 3 using EIGENSOFT v.6.1.4 [65] to provide a visual representation of the relationships between individuals and the overall structure of populations. Second, a maximum likelihood (ML) phylogenetic tree of *M. lacei* was constructed based on Dataset 2 using the GTR + GAMMA model in RAxML v.8.2.9 [66]. The resulting tree was visualized using ITOL v.6 (https://itol.embl.de/).

Since the PB population consisted of only a single individual, it was not representative for genetic diversity analysis. Therefore, for the analysis of genetic diversity and population differentiation, only populations with ≥ 4 individuals were included. Pairwise *F*_ST_ values, nucleotide diversity (π), observed heterozygosity (*H*_O_), expected heterozygosity (*H*_E_), the inbreeding coefficient (*F*_IS_), and Tajima's *D* were computed for each pairwise comparison among populations using VCFtools v.0.1.13 with Dataset 1 and Dataset 2.

To explore the impact of geographic and environmental on the distribution and genetic structure of *M. lacei*, we conducted IBD test to examine the influence of geographic distance on genetic distance connectivity between individuals. Additionally, IBE test was performed to assess the effect of environmental distance on genetic variation among individuals. The relationship between these factors was evaluated using the Mantel test, with genetic distance represented by *F*_ST_/(1-*F*_ST_) for a more accessible and comparable measure of genetic distance.

### Population history dynamics and gene flow

Stairway Plot 2 [67] is a statistical method based on genetic variation data that allows inference of population history and evolutionary processes by analyzing genetic variation within populations. The total 64 individuals were mainly divided into two lineages (KM and OTHER) based on their geographic origin, genetic differentiation and genetic structure (see above), these strongly support the separation of the KM *ex-situ* population from other populations. The analysis was conducted separately for the two lineages KM (*ex-situ* individuals propagated by seeds collected from western Yunnan province of China) and OTHER (southeastern Yunnan province of China, including PB, MLP, BZ, MC, QCT and JP populations). To infer the population history of *M. lacei*, the reference genome for *M. sinica* was used to construct the Site Frequency Spectra (SFS) of *M. lacei*, based on the “bam” files of the samples, using ANGSD v.0.921 [68]. Two types of SFS were obtained: Unfolded SFS, which considers frequency differences of all alleles and provides more detailed genetic information for population history and structure analysis, and folded SFS, which is used as a reference for convenience of calculation and analysis. The nucleotide mutation rate was estimated to be 4e-9 based on *M. sinica* as a reference. According to cultivation records from the KBG, it takes approximately 30 years for the *M. lacei* plants propagated from seeds, to reach the mature phase of flowering and fruiting [24]. These data were integrated and input into the Stairway Plot 2 script file for analysis.

TreeMix v.1.13 [43] was used to infer the amount and direction of gene flow between populations based on genetic data. Using Python v.2.7, the frequency information for the different alleles from the VCF of Dataset 2 was converted into a “freq” file and then transformed into a format suitable for TreeMix analysis. Migration events ranging from 1 to 15 were tested, and the explanatory power of each migration event was examined. Migration events with an explanatory power above 99.8% were selected as the optimal number of migration edges. A network of gene flow between populations was generated and visualized.

Outlier SNPs associated with environmental variables.

### Bioclimatic variables

Climate data from 1970–2000 were obtained from worldclim (https://www.worldclim.org/), which includes 19 bioclimatic variables [69] (Table S7). The locations of 52 individuals in the field were filtered using ArcGIS v.10.8 [70], with only one site being retained within 10 km. The filtered sites were used for extracting climate variables. Correlations were calculated among the environmental variables, and MaxEnt v.3.4.3 [71] was used for weight analysis. Variables with correlation coefficient |r|> 0.7 and low contribution were excluded, and the remaining variables were used for the next analysis. Variance Inflation Factors (VIFs) were calculated for the selected environmental factors, and all retained variables had a VIF value less than 10 [72]. Finally, four unrelated environmental variables (including contribution degree) were selected for further analysis: BIO3 (Isothermality, 4.6%), BIO7 (Temperature annual range, 77.6%), BIO13 (Precipitation of the wettest month, 3.3%), and BIO14 (Precipitation of the driest month, 4.6%) (Table S8).

### Detection of outlier SNPs

Two methods, BayeScEnv [73] and redundancy analysis (RDA) [74], were utilized to filter SNP sites related to the environment. Prior to conducting the BayeScEnv analysis, the vcf file containing SNPs was converted into GESTE/BayeScan format using PGDSpider v.2.1.1.5 [75]. The obtained environmental variables (BIO3, BIO7, BIO13, BIO14) were also standardized, and individual calculations were performed for each environmental factor with default settings. Additionally, RDA based on the vegan v.2.5.6 [74] package in R v.4.1.2 was conducted, demonstrating that the VIF values for all environmental factors were below 10, and indicating that no environmental factor needed to be discarded. To select environment-related SNPs, those falling within the tails of a ± 3 SD cutoff (two-tailed *p*-value = 0.0027) were identified as candidate SNPs. The SNPs identified by both methods were merged and then matched to the genome of *M. sinica* for candidate gene identification. Gene Ontology (GO) enrichment analysis was performed using TBtools [76] to examine the functional annotations of the corresponding genes.

### Supplementary Information

**Additional file 1: Table S1.** Detailed sampling information of seven *Michelia lacei* populations in Yunnan. **Table S2.** The CV error values of the two datasets at values of K between 2–5. **Table S3.** Measures of genetic diversity for 63 *Michelia lacei* individuals from Dataset 2. N, number of individuals in the population; π, nucleotide diversity; *H*_O_, observed heterozygosity; *H*_E_, heterozygosity within populations; *F*_IS_, inbreeding coefficient; Tajima's *D*, neutrality test statistics. **Table S4.** Genetic distances (*F*_ST_ values) between *Michelia lacei* populations based on Dataset 2. **Table S5.** Number of candidate SNP loci under putative selection identified by BAYESCENV and RDA; the VIF values of the four environmental factors. **Table S6.** GO enrichment of environment-associated genes (the combined data from the BAYESCENV and RDA analysis) in *Michelia lacei* (*P* < 0.01). **Table S7.** Detailed sampling information of 7 *Michelia lacei* populations in Yunnan. **Table S8.** Environmental variables used in this study and the contribution of four selected environmental factors to *M. lacei* distribution. **Table S9.** Correlation between the selected environmental factors. **Fig. S1.** Genetic structure obtained in Dataset 2 when K = 3. Different colors represent different genetic backgrounds. **Fig. S2.** Principal components analysis scatterplots based on the PCA results obtained from Datasets 2, where each point represents one plant. **Fig. S3.** genetic distance represented by *F*_ST_/(1-*F*_ST_) (a) IBE: genetic distance and environment distance (*R*^2^ = 0.519, *p* = 0.001), (b) IBD: genetic distance and geographical distance (*R*^2^ = 0.501, *p* = 0.001). **Fig. S4.** Population history was inferred for two *Michelia lacei* lineages (KM, OTHER) based on Folded SFS. The 95% confidence interval for the estimated effective population size is shown in light colors, and thick lines represent the median value. The light gray areas represent different glaciation events during the Pleistocene (XG, Xixiabangma Glaciation; LGP, Last Glaciation Period).

## Data Availability

The datasets presented in this study can be found in online repositories. 64 transcriptome data from this study have been submitted to NCBI (https://www.ncbi.nlm.nih.gov) and the accession number(s) can be found below: www.ncbi.nlm.nih.gov/bioproject/PRJNA1032160.

## Abbreviations

PSESP: Plant Species with Extremely Small Populations
SNPs: Single-nucleotide polymorphisms
4DTv: Fourfold degenerate synonymous site
PB: Pinbian
MLP: Malipo
BZ: Bazhai
MC: Miechang
QCT: Qincaitang
JP: Jinping
KM: Kunming
PCA: Principal component analysis
SFS: Site Frequency Spectra
MaxEnt: Maximum-entropy model
N: Number of individuals
*F*_ST_: Genetic diferentiation coefcient
π: Nucleotide diversity
*H*_O_: Observed heterozygosity
*H*_E_: Expected heterozygosity
*F*_IS_: Inbreeding coefficients
Ne: Contemporary effective population size
IBD: Isolation-by-distance
IBE: Isolation-by-environment
