# Supplementary material for: Transcriptome data analysis provides insights into the conservation of Michelia lacei, a plant species with extremely small populations distributed in Yunnan province, China
Source: BMC Plant Biol. 2024 Mar 19;24:200. doi: 10.1186/s12870-024-04892-1 (PMC10949798; doi:10.1186/s12870-024-04892-1)

**Table S1.** Raw sequencing statistics and mapping rate (with *Magnolia sinica* as the reference genome)

|  | Sample | Length(bp) | Q20(%) | Q30(%) | GC(%) | TotalReads | TotalBases | Mapping rate |
| --- | --- | --- | --- | --- | --- | --- | --- | --- |
| 1 | BZ01 | 150;150 | 97.95;98.39 | 93.58;94.66 | 46.77;46.77 | 56709190 | 8506378500 | 99.36% |
| 2 | BZ02 | 150;150 | 97.80;98.01 | 93.13;93.42 | 47.19;47.27 | 43453612 | 6518041800 | 99.21% |
| 3 | BZ03 | 150;150 | 97.66;97.98 | 92.65;93.24 | 46.96;46.96 | 54778046 | 8216706900 | 99.13% |
| 4 | BZ04 | 150;150 | 97.70;98.03 | 92.79;93.43 | 47.06;47.06 | 63837234 | 9575585100 | 99.03% |
| 5 | BZ05 | 150;150 | 97.69;98.13 | 92.82;93.82 | 47.31;47.36 | 43618818 | 6542822700 | 99.22% |
| 6 | BZ06 | 150;150 | 97.47;98.18 | 92.14;93.92 | 47.11;47.16 | 40524386 | 6078657900 | 99.27% |
| 7 | BZ07 | 150;150 | 97.97;98.37 | 93.62;94.49 | 47.70;47.71 | 49620754 | 7443113100 | 98.89% |
| 8 | BZ08 | 150;150 | 97.78;98.38 | 93.07;94.57 | 47.20;47.23 | 48445500 | 7266825000 | 99.23% |
| 9 | BZ09 | 150;150 | 97.65;98.40 | 92.71;94.65 | 47.33;47.37 | 61580654 | 9237098100 | 99.28% |
| 10 | BZ10 | 150;150 | 97.89;98.10 | 93.42;93.75 | 47.07;47.15 | 43943440 | 6591516000 | 99.00% |
| 11 | BZ11 | 150;150 | 97.50;97.95 | 92.21;93.15 | 46.92;46.99 | 41461326 | 6219198900 | 99.22% |
| 12 | BZ12 | 150;150 | 97.48;97.66 | 92.10;92.19 | 47.14;47.21 | 57069232 | 8560384800 | 99.29% |
| 13 | BZ13 | 150;150 | 97.33;98.05 | 91.65;93.30 | 46.95;47.03 | 45448222 | 6817233300 | 99.07% |
| 14 | BZ14 | 150;150 | 97.39;97.84 | 91.84;92.72 | 46.79;46.84 | 75491740 | 11323761000 | 99.31% |
| 15 | BZ15 | 150;150 | 97.62;97.98 | 92.54;93.25 | 47.02;47.07 | 52255638 | 7838345700 | 99.38% |
| 16 | BZ16 | 150;150 | 97.43;98.32 | 91.98;94.30 | 46.93;46.95 | 47978110 | 7196716500 | 99.38% |
| 17 | BZ17 | 150;150 | 97.49;97.96 | 92.17;93.14 | 47.19;47.24 | 47617670 | 7142650500 | 99.23% |
| 18 | BZ18 | 150;150 | 97.42;97.92 | 91.98;93.00 | 46.88;46.93 | 51177328 | 7676599200 | 98.75% |
| 19 | BZ19 | 150;150 | 97.42;97.80 | 91.92;92.60 | 46.84;46.91 | 43530494 | 6529574100 | 99.25% |
| 20 | BZ20 | 150;150 | 97.53;98.14 | 92.30;93.77 | 47.24;47.31 | 69263940 | 10389591000 | 99.11% |
| 21 | MC01 | 150;150 | 97.95;98.07 | 93.55;93.56 | 47.38;47.44 | 52304906 | 7845735900 | 99.24% |
| 22 | MC02 | 150;150 | 97.77;98.42 | 93.01;94.63 | 47.52;47.55 | 62684696 | 9402704400 | 98.70% |
| 23 | MLP01 | 150;150 | 97.76;98.27 | 93.04;94.23 | 47.28;47.36 | 43372348 | 6505852200 | 99.13% |
| 24 | MLP03 | 150;150 | 97.70;98.19 | 92.84;94.00 | 47.22;47.25 | 59048010 | 8857201500 | 99.23% |
| 25 | MLP04 | 150;150 | 97.71;98.22 | 92.84;94.07 | 47.04;47.07 | 57547844 | 8632176600 | 99.10% |
| 26 | MLP05 | 150;150 | 97.73;98.42 | 92.92;94.75 | 47.35;47.40 | 40161920 | 6024288000 | 99.12% |
| 27 | MLP06 | 150;150 | 97.79;98.25 | 93.11;94.21 | 47.00;47.03 | 42607620 | 6391143000 | 99.19% |
| 28 | PB01 | 150;150 | 97.86;98.51 | 93.36;95.09 | 48.31;48.28 | 57730754 | 8659613100 | 99.32% |
| 29 | JP01 | 150;150 | 96.75;98.25 | 90.82;94.13 | 47.00;47.05 | 64819624 | 9722943600 | 99.20% |
| 30 | JP02 | 150;150 | 96.95;98.24 | 91.33;94.07 | 47.07;47.15 | 49799772 | 7469965800 | 99.02% |
| 31 | JP03 | 150;150 | 96.87;98.33 | 91.16;94.37 | 47.35;47.37 | 50678166 | 7601724900 | 99.11% |
| 32 | JP04 | 150;150 | 96.34;98.48 | 89.77;94.90 | 47.18;47.17 | 79615682 | 11942352300 | 98.69% |
| 33 | KM11 | 150;150 | 97.01;98.31 | 91.53;94.31 | 47.36;47.40 | 66998124 | 10049718600 | 98.71% |
| 34 | KM12 | 150;150 | 97.16;98.36 | 91.93;94.51 | 47.03;47.07 | 64041992 | 9606298800 | 99.19% |
| 35 | QCT01 | 150;150 | 97.09;98.37 | 91.76;94.52 | 46.78;46.82 | 67628452 | 10144267800 | 99.14% |
| 36 | QCT02 | 150;150 | 96.83;98.34 | 91.07;94.43 | 46.65;46.73 | 58891172 | 8833675800 | 98.64% |
| 37 | QCT03 | 150;150 | 96.89;98.16 | 91.16;93.82 | 47.02;47.06 | 67602522 | 10140378300 | 97.34% |
| 38 | QCT04 | 150;150 | 96.70;98.19 | 90.68;93.90 | 46.83;46.90 | 58910682 | 8836602300 | 99.05% |
| 39 | QCT05 | 150;150 | 96.79;98.37 | 90.97;94.54 | 47.19;47.24 | 61453138 | 9217970700 | 99.03% |
| 40 | QCT06 | 150;150 | 97.18;98.32 | 91.98;94.34 | 47.25;47.29 | 64723814 | 9708572100 | 99.15% |
| 41 | QCT07 | 150;150 | 96.91;98.47 | 91.27;94.84 | 47.05;47.09 | 66261790 | 9939268500 | 99.07% |
| 42 | MC04 | 150;150 | 97.05;98.48 | 91.68;94.95 | 47.97;47.99 | 44198866 | 6629829900 | 99.15% |
| 43 | MC05 | 150;150 | 97.02;98.23 | 91.56;94.10 | 47.10;47.11 | 61494110 | 9224116500 | 98.94% |
| 44 | MC06 | 150;150 | 96.85;98.38 | 91.12;94.52 | 47.05;47.05 | 61983308 | 9297496200 | 99.10% |
| 45 | MC07 | 150;150 | 97.00;98.32 | 91.49;94.38 | 47.20;47.21 | 70390260 | 10558539000 | 98.94% |
| 46 | MC08 | 150;150 | 97.05;98.49 | 91.68;94.98 | 47.56;47.59 | 57454334 | 8618150100 | 99.15% |
| 47 | MC09 | 150;150 | 96.86;98.44 | 91.17;94.78 | 47.02;47.09 | 59751436 | 8962715400 | 99.29% |
| 48 | MC10 | 150;150 | 96.94;98.59 | 91.40;95.29 | 47.20;47.25 | 60456488 | 9068473200 | 99.08% |
| 49 | MC11 | 150;150 | 96.94;98.09 | 91.30;93.61 | 47.00;47.01 | 70536600 | 10580490000 | 99.20% |
| 50 | MC12 | 150;150 | 96.52;98.32 | 90.23;94.35 | 46.83;46.87 | 60721080 | 9108162000 | 99.26% |
| 51 | MC13 | 150;150 | 95.95;98.25 | 88.77;94.04 | 46.87;46.88 | 61600116 | 9240017400 | 99.23% |
| 52 | MC14 | 150;150 | 96.95;98.36 | 91.37;94.52 | 46.91;46.94 | 64047134 | 9607070100 | 98.20% |
| 53 | KM01 | 150;150 | 96.95;98.49 | 91.41;94.94 | 46.96;47.06 | 84164280 | 12624642000 | 99.25% |
| 54 | KM02 | 150;150 | 96.78;98.19 | 90.91;93.93 | 47.35;47.41 | 65521666 | 9828249900 | 99.30% |
| 55 | KM03 | 150;150 | 96.85;98.30 | 91.13;94.34 | 47.81;47.84 | 58200198 | 8730029700 | 99.16% |
| 56 | KM04 | 150;150 | 96.68;98.04 | 90.64;93.42 | 47.49;47.61 | 51535544 | 7730331600 | 99.21% |
| 57 | KM05 | 150;150 | 96.80;98.19 | 90.97;93.96 | 47.40;47.45 | 57050400 | 8557560000 | 99.29% |
| 58 | KM06 | 150;150 | 96.69;98.21 | 90.67;93.95 | 47.19;47.24 | 59077050 | 8861557500 | 99.20% |
| 59 | KM07 | 150;150 | 96.75;98.13 | 90.83;93.75 | 46.77;46.85 | 53932052 | 8089807800 | 99.23% |
| 60 | KM08 | 150;150 | 96.92;98.32 | 91.27;94.36 | 47.40;47.42 | 60076778 | 9011516700 | 99.20% |
| 61 | KM09 | 150;150 | 96.75;98.14 | 90.83;93.76 | 48.30;48.32 | 59701532 | 8955229800 | 99.17% |
| 62 | KM10 | 150;150 | 96.93;98.33 | 91.36;94.42 | 46.94;46.99 | 46837498 | 7025624700 | 99.21% |
| 63 | MC03 | 150:150 | 97.8:98.26 | 93.13:94.29 | 47.51:47.51 | 23853548 | 7156064400 | 99.32% |
| 64 | MLP02 | 150:150 | 97.77:98.22 | 93.03:94.25 | 47.22:47.20 | 26782637 | 8034791100 | 99.29% |

**Table S2.** The CV error values of the two datasets when K = 2–5.

| Dataset K | 2 | 3 | 4 | 5 |
| --- | --- | --- | --- | --- |
| Dataset 2 | 0.57766 | **0.49150** | 0.49542 | 0.51462 |
| Dataset 3 | 0.6232 | **0.60492** | 0.66497 | 0.72423 |

**Table S3.** Measures of genetic diversity for 63 *Michelia lacei* individuals from Dataset 2. N, number of individuals in the population; π, nucleotide diversity; *H*_O_, observed heterozygosity; *H*_E_, heterozygosity within populations; *F*_IS_, inbreeding coefficient; Tajima's *D*, neutrality test statistics.

| Population | N | π | *H*_O_ | *H*_E_ | *F*_IS_ | Tajima's *D* |
| --- | --- | --- | --- | --- | --- | --- |
| MLP | 6 | 0.209 | 0.314 | 0.609 | 0.169 | 0.486 |
| BZ | 20 | 0.262 | 0.353 | 0.676 | -0.087 | 0.674 |
| MC | 14 | 0.279 | 0.313 | 0.672 | 0.044 | 0.559 |
| QCT | 7 | 0.268 | 0.345 | 0.634 | 0.051 | 0.413 |
| JP | 4 | 0.269 | 0.384 | 0.583 | 0.063 | 0.269 |
| KM | 12 | 0.155 | 0.477 | 0.524 | -0.003 | 1.476 |
| Overall | 63 | 0.302 | 0.237 | 0.699 | 0.209 | 0.918 |

**Table S4.** Genetic distances (*F*_ST_ values) between *Michelia lacei* populations based on Dataset 2.

|  | MLP | BZ | MC | QCT | JP |
| --- | --- | --- | --- | --- | --- |
| BZ | 0.201 | - |  |  |  |
| MC | 0.155 | 0.099 | - |  |  |
| QCT | 0.166 | 0.113 | 0.042 | - |  |
| JP | 0.152 | 0.114 | 0.048 | 0.053 | - |
| KM | 0.384 | 0.232 | 0.215 | 0.268 | 0.306 |

**Table S5.** Number of candidate SNP loci under putative selection identified by BAYESCENV and RDA; the VIF values of the four environmental factors.

| Method | SNPs | BIO3 | BIO7 | BIO13 | BIO14 |
| --- | --- | --- | --- | --- | --- |
| BAYESCENV | 173 | 52 | 46 | 15 | 129 |
| RDA | 636 | 168 | 248 | 112 | 108 |
| VIF | - | 3.79 | 3.49 | 1.48 | 2.49 |

Note: BIO3 (Isothermality), BIO7 (Temperature Annual Range), BIO13 (Precipitation of the Wettest Month), and BIO14 (Precipitation of the Driest Month)

**Table S6.** GO enrichment of environment associated genes (the union data of BAYESCENV and RDA) of *Michelia lacei* (*P* < 0.01).

| GO.ID | Term | Class | Related gene | P_value |
| --- | --- | --- | --- | --- |
| GO:0005102 | signaling receptor binding | Molecular function | 9 | 0.005508157 |
| GO:1901981 | phosphatidylinositol phosphate binding | Molecular function | 5 | 0.008024706 |
| GO:0120038 | obsolete plasma membrane bounded cell projection part | Cellular component | 7 | 3.68E-04 |
| GO:0044463 | obsolete cell projection part | Cellular component | 7 | 3.68E-04 |
| GO:0099568 | cytoplasmic region | Cellular component | 10 | 0.005797658 |
| GO:0051641 | cellular localization | Biological process | 42 | 0.00214135 |
| GO:0008104 | protein localization | Biological process | 28 | 0.003208721 |
| GO:0046907 | intracellular transport | Biological process | 29 | 0.003239562 |
| GO:0070925 | organelle assembly | Biological process | 13 | 0.004295392 |
| GO:0070727 | cellular macromolecule localization | Biological process | 30 | 0.004897694 |
| GO:0022607 | cellular component assembly | Biological process | 35 | 0.005239234 |
| GO:0033036 | macromolecule localization | Biological process | 33 | 0.005438438 |
| GO:0019751 | polyol metabolic process | Biological process | 5 | 0.00567455 |
| GO:0006886 | intracellular protein transport | Biological process | 20 | 0.006630434 |
| GO:0035195 | miRNA-mediated post-transcriptional gene silencing | Biological process | 5 | 0.006895473 |
| GO:0071236 | cellular response to antibiotic | Biological process | 5 | 0.008289082 |
| GO:0006066 | alcohol metabolic process | Biological process | 9 | 0.009712345 |
| GO:0044275 | obsolete cellular carbohydrate catabolic process | Biological process | 6 | 0.009752688 |

**Table S7.** Detailed sampling information of 7 *Michelia lacei* populations in Yunnan.

| Code | Locality | Longitude | Latitude | Altitude (m) | Sample Size | Voucher Code |
| --- | --- | --- | --- | --- | --- | --- |
| PB | Pinbian | 103.8871 | 23.0519 | 1580 | 1 | YL2022001 |
| MLP | Malipo | 104.7046 | 23.0677 | 1357 | 6 | YL2022002 |
| BZ | Bazhai | 104.0621 | 23.0045 | 1493 | 20 | YL2022003 |
| MC | Miechang | 104.0953 | 22.9121 | 1490 | 14 | YL2022004 |
| QCT | Qincaitang | 104.0267 | 22.6665 | 1195 | 7 | YL2022005 |
| JP | Jinping | 103.1650 | 22.9589 | 1346 | 4 | YL2022006 |
| KM | Kunming | 102.7429 | 25.1382 | 1924 | 12 | YL2022007 |

**Table S8.** Environmental variables used in this study and the contribution of four selected environmental factors to *M. lacei* distribution

| Name | Description | Contribution degree |
| --- | --- | --- |
| BIO1 | Annual Mean Temperature |  |
| BIO2 | Mean Diurnal Range |  |
| BIO3 | Isothermality | **4.6%** |
| BIO4 | Temperature Seasonality |  |
| BIO5 | Max Temperature of the Warmest Month |  |
| BIO6 | Min Temperature of the Warmest Month |  |
| BIO7 | Temperature Annual Range | **77.6%** |
| BIO8 | Mean Temperature of the Wettest Quarter |  |
| BIO9 | Mean Temperature of the Driest Quarter |  |
| BIO10 | Mean Temperature of the Warmest Quarter |  |
| BIO11 | Mean Temperature of the Coldest Quarter |  |
| BIO12 | Annual Precipitation |  |
| BIO13 | Precipitation of the Wettest Month | **3.3%** |
| BIO14 | Precipitation of the Driest Month | **4.6%** |
| BIO15 | Precipitation Seasonality |  |
| BIO16 | Precipitation of the Wettest Quarter |  |
| BIO17 | Precipitation of the Driest Quarter |  |
| BIO18 | Precipitation of the Warmest Quarter |  |
| BIO19 | Precipitation of the Coldest Quarter |  |

**Table S9.** Correlation between the selected environmental factors.

|  | BIO3 | BIO7 | BIO13 | BIO14 |
| --- | --- | --- | --- | --- |
| BIO3 | 1 | -0.6037 | -0.5430 | -0.3062 |
| BIO7 | -0.6037 | 1 | 0.4417 | -0.3779 |
| BIO13 | -0.5430 | 0.4417 | 1 | -0.0006 |
| BIO14 | -0.3062 | -0.3779 | -0.0006 | 1 |

**Fig. S1.** Genetic structure obtained in Dataset 2 when K=3. Different colors represent different genetic backgrounds.

**
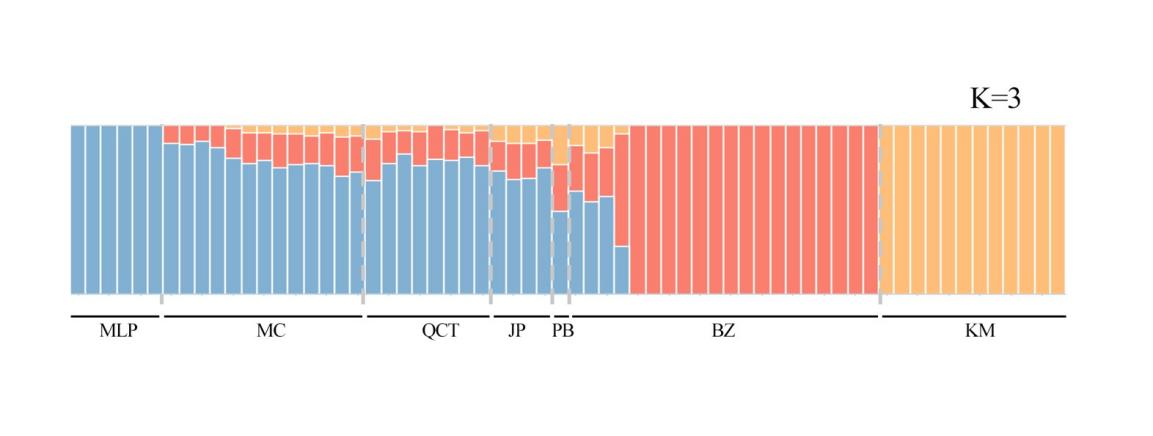
**

**Fig. S2.** Principal components analysis scatterplots based on the PCA results obtained from Datasets 2, where each point represents one plant.

**
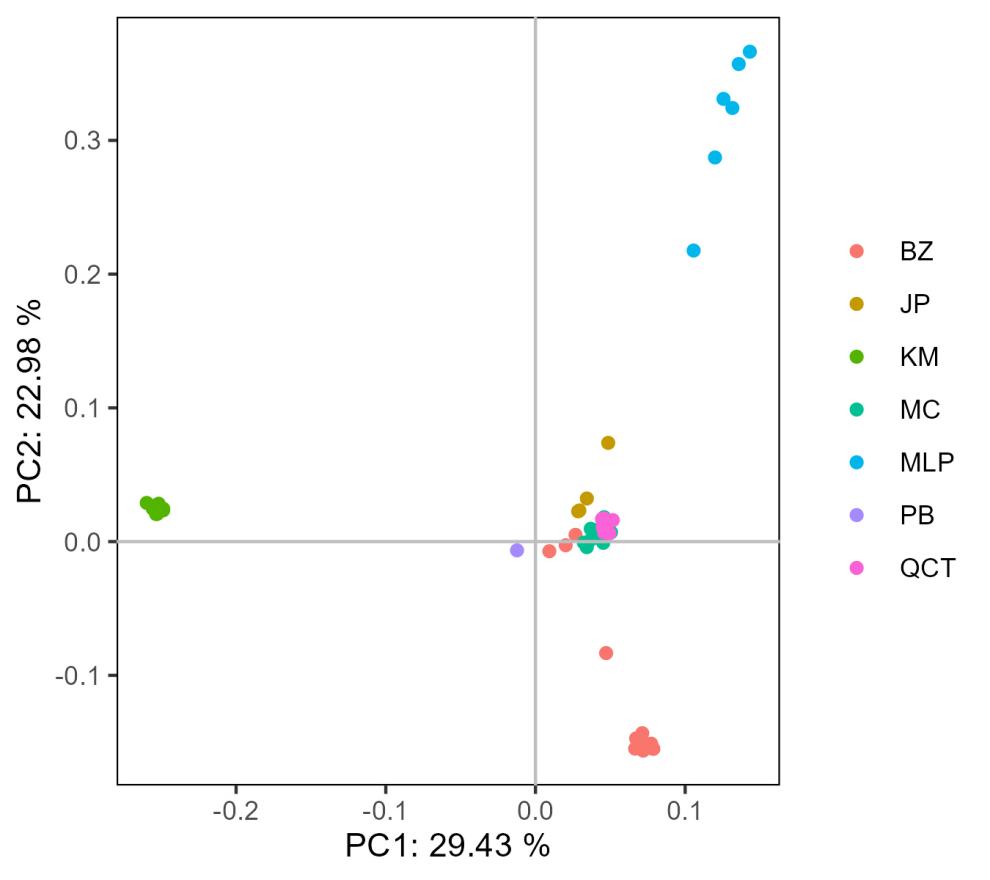
**

**Fig. S3.** genetic distance represented by *F*_ST_/(1-*F*_ST_) **(a)** IBE: genetic distance and environment distance (R^2^ = 0.519, p = 0.001), **(b)** IBD: genetic distance and geographical distance (R^2^ = 0.501, p = 0.001).

**
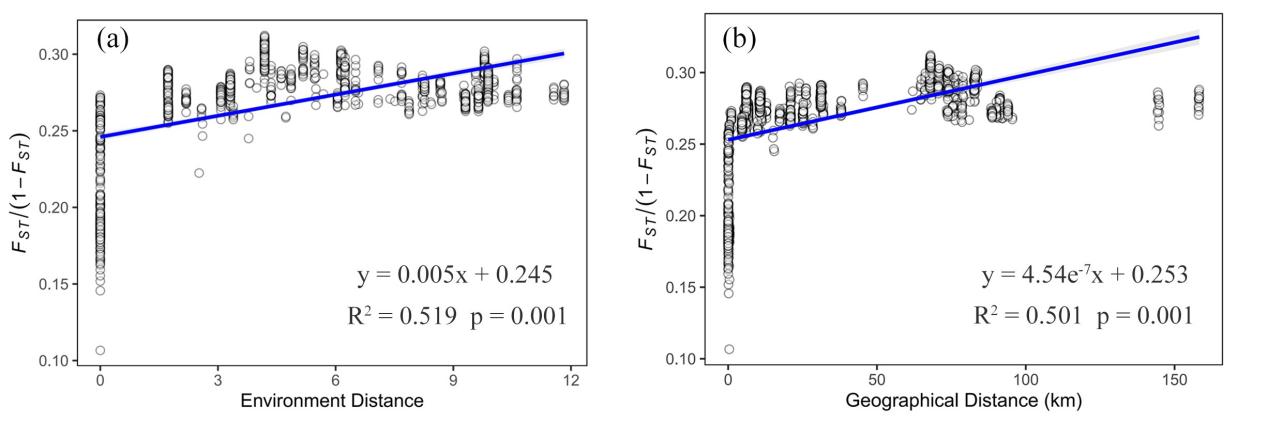
**

**Fig. S4.** Population history was inferred for two *Michelia lacei* lineages (KM, OTHER) based on Folded SFS. The 95% confidence interval for the estimated effective population size is shown by light color, thick lines represent the median. The light grey areas represent different glaciation events during the Pleistocene (XG, Xixiabangma Glaciation; LGP, Last Glaciation Period).


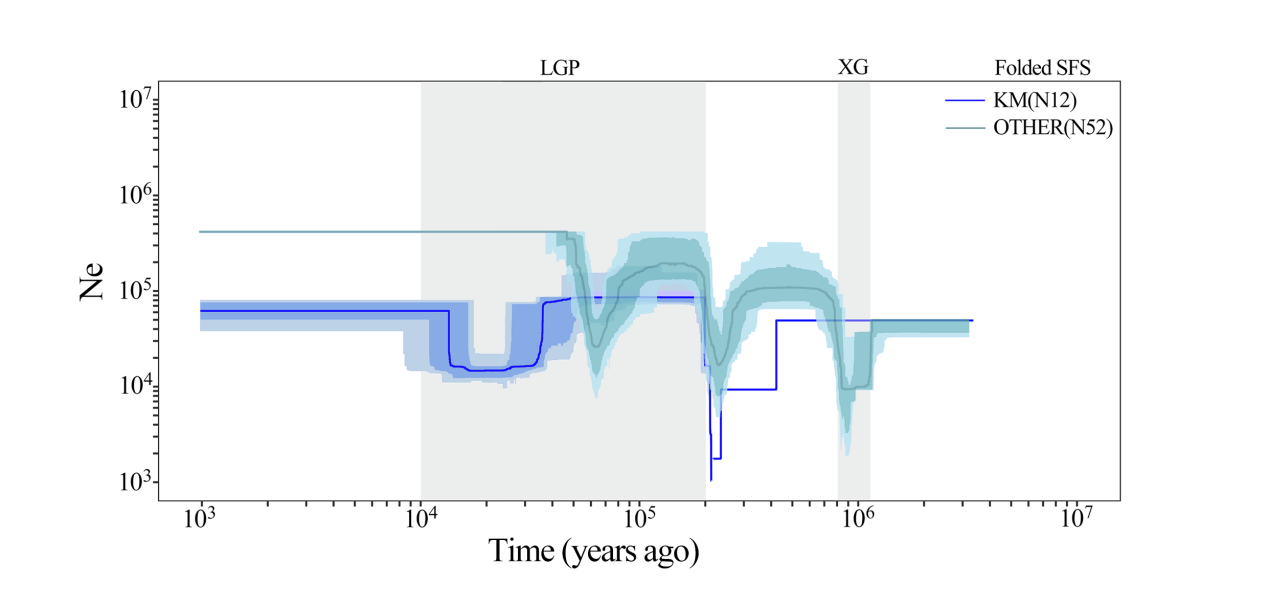

Supplement: Supplementary file 1 — Additional file 1: Table S1. Detailed sampling information of seven Michelia lacei populations in Yunnan. Table S2. The CV error values of the two datasets at values of K between 2–5. Table S3. Measures of genetic diversity for 63 Michelia lacei individuals from Dataset 2. N, number of individuals in the population; π, nucleotide diversity; HO, observed heterozygosity; HE, heterozygosity within populations; FIS, inbreeding coefficient; Tajima's D, neutrality test statistics. Table S4. Genetic distances (FST values) between Michelia lacei populations based on Dataset 2. Table S5. Number of candidate SNP loci under putative selection identified by BAYESCENV and RDA; the VIF values of the four environmental factors. Table S6. GO enrichment of environment-associated genes (the combined data from the BAYESCENV and RDA analysis) in Michelia lacei (P < 0.01). Table S7. Detailed sampling information of 7 Michelia lacei populations in Yunnan. Table S8. Environmental variables used in this study and the contribution of four selected environmental factors to M. lacei distribution. Table S9. Correlation between the selected environmental factors. Fig. S1. Genetic structure obtained in Dataset 2 when K = 3. Different colors represent different genetic backgrounds. Fig. S2. Principal components analysis scatterplots based on the PCA results obtained from Datasets 2, where each point represents one plant. Fig. S3. genetic distance represented by FST/(1-FST) (a) IBE: genetic distance and environment distance (R2 = 0.519, p = 0.001), (b) IBD: genetic distance and geographical distance (R2 = 0.501, p = 0.001). Fig. S4. Population history was inferred for two Michelia lacei lineages (KM, OTHER) based on Folded SFS. The 95% confidence interval for the estimated effective population size is shown in light colors, and thick lines represent the median value. The light gray areas represent different glaciation events during the Pleistocene (XG, Xixiabangma Glaciation; LGP, Last Glaciation [file 12870_2024_4892_MOESM1_ESM.doc]
